# Supplementary material for: Exploring the genetic diversity and population structure of aerial yams (Dioscorea bulbifera L.) DArT-seq and agronomic traits
Source: PLoS One. 2024 Aug 23;19(8):e0306631. doi: 10.1371/journal.pone.0306631 (PMC11343425; doi:10.1371/journal.pone.0306631)
Supplement: Attachment — Submitted filename: PONE-D-23-28562 (1).pdf [file pone.0306631.s001.pdf]

PLOS ONE

Exploring the Genetic Diversity and Population Structure of Aerial yams (Dioscorea bulbifera L.) DArT-seq and agronomic traits.

--Manuscript Draft--

**Manuscript Number:**	PONE-D-23-28562
**Article Type:**	Research Article
**Full Title:**	Exploring the Genetic Diversity and Population Structure of Aerial yams (Dioscorea bulbifera L.) DArT-seq and agronomic traits.
**Short Title:**	Exploring the Genetic Diversity of Aerial yams (Dioscorea bulbifera L.) in Nigeria
**Corresponding Author:**	Paterne A. Agre, Ph.D<br>International Institute of Tropical Agriculture<br>Ibadan, Oyo NIGERIA
**Keywords:**	: Dioscorea bulbifera; population structure; SNP markers; Nigeria
**Abstract:**	Dioscorea bulbifera is an edible yam specie with aerial bulbils. Assessing the genetic diversity of D. bulbifera accession for cultivation and breeding purposes is essential for it genetic improvement, especially where the crop faces minimal attention. The aims of this study was to assess the genetic diversity of Dioscorea bulbifera accessions collected from Nigeria and accessions maintained at the genebank of International Institute of Tropical Agriculture (IITA) Ibadan. Accessions were profiled using quatitative traits and sequenced using the Diversity Array Technology)DArTseq). Multivariate analysis based phenotypic traits revealed high variability among the evaluated accessions and all phenotypic traits assessed were useful in discriminating the aerial yam accessions. Using DArT-Seq marker, the 94 accessions were classified into three genetic group through the admixture and the phylogeny analysis. The analysis of molecular variance (AMOVA) displayed low genetic diversity within genetic group with the highest genetic diversity been observed among the genetic group. The study established a baseline for the selection of parental lines from the genetic groups for genetic improvement of the D. bulbifera.
**Order of Authors:**	Paterne A. Agre, Ph.D
	Eunice Ekaette
	Emeka Nwofia
	Peter Okocha
	Ikenna Nnnabue
	Kenneth Eluwa
	Jude Obidiegwu
**Additional Information:**	
**Question**	**Response**
**Financial Disclosure**<br><br>Enter a financial disclosure statement that describes the sources of funding for the work included in this submission. Review the submission guidelines for detailed requirements. View published research articles from *PLOS ONE* for specific examples.<br><br>This statement is required for submission and **will appear in the published article** if	Include this sentence at the end of your statement: The funders had no role in study design, data collection and analysis, decision to publish, or preparation of the manuscript.

Powered by Editorial Manager® and ProduXion Manager® from Aries Systems Corporation

the submission is accepted. Please make sure it is accurate.<br><br>**Unfunded studies**<br>Enter: *The author(s) received no specific funding for this work.*<br><br>**Funded studies**<br>Enter a statement with the following details:<br>• Initials of the authors who received each award<br>• Grant numbers awarded to each author<br>• The full name of each funder<br>• URL of each funder website<br>• Did the sponsors or funders play any role in the study design, data collection and analysis, decision to publish, or preparation of the manuscript?<br>• **NO** - Include this sentence at the end of your statement: *The funders had no role in study design, data collection and analysis, decision to publish, or preparation of the manuscript.*<br>• **YES** - Specify the role(s) played.<br><br>* typeset	
**Competing Interests**<br><br>Use the instructions below to enter a competing interest statement for this submission. On behalf of all authors, disclose any competing interests that could be perceived to bias this work—acknowledging all financial support and any other relevant financial or non-financial competing interests.<br><br>This statement is required for submission and **will appear in the published article** if the submission is accepted. Please make sure it is accurate and that any funding sources listed in your Funding Information later in the submission form are also declared in your Financial Disclosure statement.<br><br>View published research articles from *PLOS ONE* for specific examples.	The authors have declared that no competing interests exist

**NO authors have competing interests**<br><br>Enter: *The authors have declared that no competing interests exist.*<br><br>**Authors with competing interests**<br><br>Enter competing interest details beginning with this statement:<br><br>*I have read the journal's policy and the authors of this manuscript have the following competing interests: [insert competing interests here]*<br><br>* typeset	
**Ethics Statement**<br><br>Enter an ethics statement for this submission. This statement is required if the study involved:<br><br>• Human participants<br>• Human specimens or tissue<br>• Vertebrate animals or cephalopods<br>• Vertebrate embryos or tissues<br>• Field research<br><br>Write "N/A" if the submission does not require an ethics statement.<br><br>General guidance is provided below. Consult the submission guidelines for detailed instructions. **Make sure that all information entered here is included in the Methods section of the manuscript.**	The Research was Conducted based on the Umudike guideline. No Human sample was collected and all collected data was based on developed SOP. The Genetic materials used here are from the IITA Genebank and was collected for research purpose only.

Powered by Editorial Manager® and ProduXion Manager® from Aries Systems Corporation

**Format for specific study types**<br><br>**Human Subject Research (involving human participants and/or tissue)**<br>• Give the name of the institutional review board or ethics committee that approved the study<br>• Include the approval number and/or a statement indicating approval of this research<br>• Indicate the form of consent obtained (written/oral) or the reason that consent was not obtained (e.g. the data were analyzed anonymously)<br><br>**Animal Research (involving vertebrate animals, embryos or tissues)**<br>• Provide the name of the Institutional Animal Care and Use Committee (IACUC) or other relevant ethics board that reviewed the study protocol, and indicate whether they approved this research or granted a formal waiver of ethical approval<br>• Include an approval number if one was obtained<br>• If the study involved *non-human primates*, add *additional details* about animal welfare and steps taken to ameliorate suffering<br>• If anesthesia, euthanasia, or any kind of animal sacrifice is part of the study, include briefly which substances and/or methods were applied<br><br>**Field Research**<br><br>Include the following details if this study involves the collection of plant, animal, or other materials from a natural setting:<br>• Field permit number<br>• Name of the institution or relevant body that granted permission	
**Data Availability**<br><br>Authors are required to make all data underlying the findings described fully available, without restriction, and from the time of publication. PLOS allows rare exceptions to address legal and ethical concerns. See the PLOS Data Policy and FAQ for detailed information.	Yes - all data are fully available without restriction

A Data Availability Statement describing where the data can be found is required at submission. Your answers to this question constitute the Data Availability Statement and **will be published in the article**, if accepted.<br><br>**Important:** Stating 'data available on request from the author' is not sufficient. If your data are only available upon request, select 'No' for the first question and explain your exceptional situation in the text box.<br><br>Do the authors confirm that all data underlying the findings described in their manuscript are fully available without restriction?	
**Describe where the data may be found in full sentences. If you are copying our sample text, replace any instances of XXX with the appropriate details.**<br><br>• If the data are **held or will be held in a public repository**, include URLs, accession numbers or DOIs. If this information will only be available after acceptance, indicate this by ticking the box below. For example: *All XXX files are available from the XXX database (accession number(s) XXX, XXX.).*<br>• If the data are all contained **within the manuscript and/or Supporting Information files**, enter the following: *All relevant data are within the manuscript and its Supporting Information files.*<br>• If neither of these applies but you are able to provide **details of access elsewhere**, with or without limitations, please do so. For example:<br><br>*Data cannot be shared publicly because of [XXX]. Data are available from the XXX Institutional Data Access / Ethics Committee (contact via XXX) for researchers who meet the criteria for access to confidential data.*<br><br>*The data underlying the results presented in the study are available from (include the name of the third party*	All data can be downloaded from the yam base using the following link www.yambase.org

and contact information or URL).<br>• This text is appropriate if the data are owned by a third party and authors do not have permission to share the data.<br><br>* typeset	
Additional data availability information:	

Manuscript Click here to access/download;Manuscript;Resived_Bulfera manuscript.290823_Submitted.docx

Exploring the Genetic Diversity and Population Structure of Aerial yams (*Dioscorea bulbifera L.*) DArT-seq and agronomic traits.

Eunice Ekaette[12], Emeka Nwofia[1], Peter Okocha[1], Ikenna Nnnabue[3], Kenneth Eluwa[3], Jude Obidiegwu[3], Paterne A. Agre[4*]

[1]Department of Agronomy, Michael Okpara University of Agriculture
[2]National Biotechnology Development Agency, Lugbe, Abuja, Nigeria
[3]National Root Crops Research Institute, Umudike, Abia State, Nigeria
[4]International Institute of Tropical Agriculture (IITA), Ibadan, Nigeria

*Correspoding author: p.agre@cgiar.org

ABSTRACT

Dioscorea bulbifera is an edible yam specie with aerial bulbils. Assessing the genetic diversity of *D. bulbifera* accession for cultivation and breeding purposes is essential for it genetic improvement, especially where the crop faces minimal attention. The aims of this study was to assess the genetic diversity of *Dioscorea bulbifera* accessions collected from Nigeria and accessions maintained at the genebank of International Institute of Tropical Agriculture (IITA) Ibadan. Accessions were profiled using quatitative traits and sequenced using the Diversity Array Technology)DArTseq). Multivariate analysis based phenotypic traits revealed high variability among the evaluated accessions and all phenotypic traits assessed were useful in discriminating the aerial yam accessions. Using DArT-Seq marker, the 94 accessions were classified into three genetic group through the admixture and the phylogeny analysis. The analysis of molecular variance (AMOVA) displayed low genetic diversity within genetic group with the highest genetic diversity been observed among the genetic group. The study established a baseline for the selection of parental lines from the genetic groups for genetic improvement of the *D. bulbifera*.

Keywords*: Dioscorea bulbifera*, population structure, SNP markers, Nigeria

INTRODUCTION

Yams (*Dioscorea* L.) belong to the family *Dioscoreaceae*. *Dioscorea* L. is the largest genus of the family *Dioscoreaceae* [1,2]. Yams (*Dioscorea* spp) are popular staples in West Africa [3] while serving as important source of dietary calories thus contributing on average, more than 200 Kilocalories per person per day to over 300 million people between 2006 and 2010 [4]. Its wide adaptability across the diverse agro-ecologies of the country and the ability of farmers to harvest their tubers when convenient, makes it an essential food security crop [5]. For millions of people, yam tubers form an integral part of social-cultural activities [6] and medicinal values [7]. Nigeria ranks as the leading producer of yams ~~(*Dioscorea* spp.)~~ in the world, accounting for 65% (about 50.1 million tons) of annual global production [8]. Though yams are grown for their carbohydrate content, their storage organs are important sources of proteins, fats and vitamins for millions of people in West Africa, Southeast Asia, and the Caribbean [9,10]. Some species are only marginally inferior to maize and rice in protein value [11]. Many species also contain substantial amounts of vitamins such as carotene, thiamine, riboflavin, niacin and some minerals like calcium, phosphorus and iron [12,13,7]. There are over 600 species of yams world wide, of which 50–60 are cultivated or gathered for food or medicinal purposes. However, there are only about 12 species of economic significance as food plants [14,15].

Dioscorea bulbifera (commonly known as the air potato, air yam, bitter yam) is native to Africa and Asia with Asian cultivars less angular, more spherical, and less toxic than African cultivars [5]. *D. bulbifera* has been therapeutic potentials in the management of ailments [7,16]. The cultivation of *D.bulbifera* in Nigeria has remained traditional and is usually carried out by smallholder farmers.The lack of adequate information on genetic diversity limits its genetic improvement. In order to develop elite genotypes that combine high and stable yield of good quality tubers as well as resistance to pests and diseases, a wide range of genetic diversity is required. Assessment of the diversity in *D. alata*, *D. cayenensis* and *D. rotundata* would enable the identification of genotypes with complementary characteristics that could serve as potential parents in breeding programmes. Studies using agro-morphological do not demonstrate the true genetic relatedness of the accessions and are strongly influenced by environmental factors [17,18]. In Nigeria, there is limited information on genetic diversity status of *D. bulbifera* using

molecular markers. Molecular markers have been utilized as powerful tools for the estimation of genetic diversity of many species with great success and accuracy because they are abundant and unaffected by environmental parameters [19]. So far, markers used for assessing diversity in yams include restriction fragment length polymorphism (RFLP) of cpDNA [20], random amplified polymorphic DNA (RAPD) [21,22] amplified fragment length polymorphism (AFLP) markers [23,24], and SSR markers [25-28]. Next-generation sequencing (DArTseq-based) has been successfully applied in germplasm characterization in white guinea yam [29-32] water yam [33-34], and in trifoliate yam [35], thus demonstrating its suitability for the high-throughput genotyping in yams. This study is aimed towards a better understanding of the genetic relationships and population structure of *D. bulbifera* in Nigeria. This will set the foundation for the crop improvement and development of breeding strategies in Nigeria

Materials and methods

Experimental site

The experiment was conducted ~~in the field~~ during the 2020 and 2021 planting seasons at the eastern farm of the National Root Crops Research Institute, Umudike, located at 5°20’ N latitude and 07°33’E longitude. Here, the annual rainfall ranges from 1800 to 2200 mm with an average annual air temperature and relative humidity of 20.50°C and 76.80%, respectively.

Collection of planting materials

The planting materials used for this study consisted of 94 *D. bulbifera* accessions representing originated from different source. A total of 64 accessions were sourced from the genebank of International Institute of Tropical Agriculture (IITA) Ibadan, Nigeria. Thirty collections were

also sourced from the National Root Crops Research Institute (NRCRI), Umudike, Nigeria (Table 1). The accessions were collected for research pupose only as agreed by the (NRCRI). The accessions were profiled for phenotypic traits and no extraits was tested on human.

Table 1: List of *Dioscorea bulbifera* accessions, their origins and agro-ecological zones

Serial No	Accession	Origin	Agro-ecological Zone
1	TDb/3069	Togo	Savannah
2	TDb/3063	Togo	Savannah
3	TDb/3694	Congo	Savannah woodland
4	TDb/3059	Togo	Savannah
5	TDb/3073	Togo	Savannah
6	TDb/4119	Guinea	Savannah
7	TDb/3119	Togo	Savannah
8	TDb/3082	Nigeria	Forest
9	TDb/3091	Gabon	Forest
10	TDb /3833	Burkina Faso	Savannah
11	TDb/3058	Togo	Savannah
12	TDb/3688	Nigeria	Forest
13	TDb/3080	Ghana	Forest
14	TDb/3065	Togo	Savannah
15	TDb/3096	Togo	Savannah
16	TDb/3693	Congo	Forest
17	TDb/3081	Gabon	Forest
18	TDb/3070	Togo	Savannah
19	TDb/4123	Sierra Leone	Savannah
20	TDb/3060	Togo	Savannah
21	TDb/3834	Nigeria	Forest
22	TDb/1455	Gabon	Forest
23	TDb/3690	Nigeria	Forest
24	TDb/3048	Benin	Savannah
25	TDb/3068	Togo	Savannah
26	TDb/3835	Nigeria	Swampy with tall tree and grasses
27	TDb/3431	Nigeria	Forest
28	TDb/3049	Benin	Savannah
29	TDb/3697	Congo	Forest
30	TDb/3769	Nigeria	Forest
31	TDb/3691	Congo	Savannah woodland

32	TDb/3072	Nigeria	Forest
33	TDb/3071	Togo	Savannah
34	TDb/3045	Nigeria	Forest
35	TDb/3047	Benin	Savannah
36	TDb/3087	Gabon	Forest
37	TDb/2857	Equatorial Guinea	Forest
38	TDb/3078	Nigeria	Forest
39	TDb/3083	Gabon	Savannah woodland
40	TDb/3695	Congo	Savanah woodland
41	TDb/3066	Togo	Savannah
42	TDb/3076	Togo	Savannah
43	TDb/4121	Sierra Leone	Savannah
44	TDb/3061	Togo	Savannah
45	TDb/3085	Nigeria	Forest
46	TDb/3088	Gabon	Forest
47	TDb/3062	Gabon	Forest
48	TDb/4120	Sierra Leone	Savannah
49	TDb/3044	Nigeria	Forest
50	TDb/3090	Gabon	Savannah woodland
51	TDb/3092	Gabon	Savannah woodland
52	TDb/4641	Togo	Savannah
53	TDb/3086	Gabon	Forest
54	TDb/3773	Nigeria	Forest
55	TDb/3079	Ghana	Forest
56	TDb/3190	Nigeria	Forest
57	TDb/3689	Nigeria	Forest
58	TDb /3091	Gabon	Forest
59	TDb/3692	Congo	Forest
60	TDb/3046	Nigeria	Forest
61	TDb/3832	Burkina Faso	Savannah
62	TDb/3067	Togo	Savannah
63	TDb/3075	Togo	Savannah
64	TDb/4122	Sierra Leone	Savannah
65	YA1	Nigeria	Forest
66	YA2	Nigeria	Forest
67	YA3	Nigeria	Forest
68	YA4	Nigeria	Forest
69	YA5	Nigeria	Forest
70	YB1	Nigeria	Forest
71	YB2	Nigeria	Forest
72	YB3	Nigeria	Forest
73	YB4	Nigeria	Forest

74	YB5	Nigeria	Forest
75	YC1	Nigeria	Forest
76	YC2	Nigeria	Forest
77	YC3	Nigeria	Forest
78	YC4	Nigeria	Forest
79	YC5	Nigeria	Forest
80	YD1	Nigeria	Forest
81	YD2	Nigeria	Forest
82	YD3	Nigeria	Forest
83	YD4	Nigeria	Forest
84	YD5	Nigeria	Forest
85	YE1	Nigeria	Forest
86	YE2	Nigeria	Forest
87	YE3	Nigeria	Forest
88	YE4	Nigeria	Forest
89	YE5	Nigeria	Forest
90	YF1	Nigeria	Forest
91	YF2	Nigeria	Forest
92	YF3	Nigeria	Forest
93	YF4	Nigeria	Forest
94	YF5	Nigeria	Forest

Field establishment

The experimental design used was the [illegible]ndomized complete block design (RCBD). The planting was arranged in ridges with planting at the beginning of the rainy season. A single row plot was used for each accession, each row measuring 7m long with 1m spacing between rows and 1m spaces between plants within a row. This spacing regimen was done to avoid competition among neighbouring plants and to ensure sound establishment of each accession. Individual plants were supported by bamboo stakes. Standard weeding and agronomic practices was regularly applied to provide plants with adequate growing conditions. The middle five healthy plants from each row was used for data collection. ~~A total of 7 quantitative morphological descriptors for yam was used to profile the 94 accessions.~~

Phenotyping

The quantitative parameters as shown in Table 2 were used to assess the agronomic performance of the 94 yam accessions. Data was recorded using the yam standard operational protocol developed by [36] with slight modification.

Table 2: Morphological descriptors used for phenotypic characterization of *D.bulbifera* yam accessions

Descriptors	Description
Vine length (m)	Measured in meters from the bottom of the plant to the last leaf
Leaf length (cm)	Measured in cm (three yam leaf were selected and measured)
Leaf width (cm)	Measured in cm (three yam leaf were selected and measured)
Petiole length (cm)	Measured in cm (three petiole were selected and measured)
Number of leaves (No)	Counting of the number of leaves of each plant
Number of branches on main stem (No)	Counting of the number of branches on the main stem of each plant
Number of stem per plant (No)	Counting of the number of leaves of each plant
Stem diameter (mm)	Measured at a distance of 15cm from the base of the plant using vernier calipers.

Source: [36];

Genotyping

Yam leaf samplingand DNA extraction

Young, healthy and fully expanded fresh leaf samples were harvested from each accession in the field. About three to five tender leaves, weighing more than 500mg to 900mg were collected in well labelled bags containing silica-gel granules with a colour indicator. The leaf samples were stored in the silica-gel for 72 hours to remove the moisture. Subsequently, genomic DNA

(gDNA) extraction was carried out at the Bioscience centre, International Institute of Tropical Agriculture (IITA), Ibadan, Nigeria, using the CTAB procedure with slight modification [37]. The DNA quality and concentration was ascertained by running the gDNA in a 1% agarose gel and on a NanoDrop 2000 spectrophotometer, following the methods described in [38].

SNP genotyping assay and SNP filtering

High quality DNA was sent to DArT Australia for the sequencing. For the sequencing-based DArT genotyping, SNPs were called using complexity reduction methods optimized for yam at the DArT's proprietary software, DArTSoft, as described by [39]. Reads and tags found in each sequencing result were aligned to the yam reference genome [40].

For quality control, the 11,721 DArTseq SNP-derived markers were filtered to eliminate the unwanted SNP markers using the software PLINK [41] version 1.9 and VCFtools [42]. Markers and genotypes with high missing value (>20%) were eliminated as well as SNP marker with low minor allele frequency (<5%) and low call rate. After quality filtering, 10,087 DArT-SNP markers distributed across 20 yams chromosomes were used for subsequent analysis.

Phenotypic data analysis

Quantitative data collected were subjected to descriptive statistical analysis (minimum, maximum, average and standard error) using basic function in R. Principal component analysis (PCA) was performed using FactorMiner package [43]. The PCA data was used to generate eigenvalues, cumulative variability, and load coefficient values. The principal components (PC) with eigenvalues > 1.0 were selected, and those traits that had load coefficients ≥ 0.5 were considered relevant scores for the PC and considered as valuable traits for distinguishing between the genotypes [44].

Assessment of genetic diversity

The filtered SNP data were used to assess the population structure and genetic diversity in aerial yam. Genetic diversity analysis was carried out using parameters such as minor allele frequency (MAF), polymorphic information content (PIC), expected heterozygosity (He), and observed heterozygosity (Ho). These were estimated using VCFtools [42] and PLINK 1.9 [41]. First, the optimal number of clusters was inferred using k-means analysis [45,46] of PCA (principal component analysis)-transformed genome-wide SNP data by varying the possible number of clusters from 1 to 100. Bayesian Information Criterion (BIC) was used to assess the best supported model as well as the number and nature of clusters. DAPC scatter plots were later developed on the clusters identified through k-means using the first 50 principal components. The information based on ADMIXTURE analysis was used to determine the most appropriate K, and accessions with membership proportions (Q-value) ≥ 60% were assigned to groups, while those with membership probabilities less than 60% were declared as admixtures [47]. For the HC analysis, generated Jaccard dissimilarity matrix was used and the HC was plotted using Ward.D2 method.

Results

Variability based phenotypic data

The result of the phenotypic statistics, including mean, minimum and maximum are shown in Table 3. High phenotypic variation was observed for quantitative traits. The accessions exhibited significant variability for most of the traits assessed. Traits such as vine length ranged from 0.87m to 3.47m with an average value of 1.85m while the number of branches on main stem ranged from 3 to 24 with 8.74 as average value. Similarly. For the yam leaf length, it ranged from 7.20 to 18.12cm among the accession with a mean value of 12.63.

Table 3: Descriptive statistics of some quantitative morphological traits of *D. bulbifera*

Phenotypic Traits	Mean ± SE	Maximum	Minimum
VL[a]	1.85 ± 0.32	3.47	0.87
Sdia[b]	3.30 ± 0.48	5.69	1.89
NL[c]	60.60 ±14.37	123.00	20.00
LL[d]	12.63 ± 1.44	18.12	7.20
Lwidth[e]	9.91 ± 1.28	17.60	2.62
NBMS[f]	8.74 ± 1.70	24.00	3.00
SPP[g]	6.00 ± 0.95	3.00	1.00

[a]Vine length (m) ; [b]Stem diameter (mm); [c]Number of leaves; [d]Leaf length (cm); [e]Leaf width (cm); [f]Number of branches on main stem; ; [g]Number of stem per plant; [h]Petiole length (cm)

Relative contribution of individual traits to phenotypic variation.

Through the PCA, the first three components explained 72.17% of the total variation (Table 4). The first principal component (PC1) had an Eigen value of 2.73, contributing to 38.94% of the total variation. On the first component, vine length, number of branches on main stem, leaf width and number of stems per plant were the major contributors to the total variation. Most of the variables were positively correlated except for stem diameter and leaf length. The second component explained 19.24% of the total variance, with major contribution from stem diameter, and number of leaves of plant. The third principal component accounted for 13.99% of the total variation with leaf length as the only trait associated with this component.

The influence of the traits on the principal components and the levels of correlation among them are presented in Figure 1. Traits like vine length (VL), number of branches on main stem, stem number per plant were found to be positively correlated on the x axis as well as number of leaves (NL),leaf length (LL) and number of leaves (NL) positively correlated on the y axis.

Table 4: The proportion of the morphological variation and traits contribution explained by the first three (3) principal components.

Traits	PC1[a]	PC2	PC3
VL[a]	**0.83**	0.17	0.09
Sdia[b]	-0.24	**-0.71**	-0.10
NL[c]	0.24	**0.71**	-0.31
NBMS[d]	**0.86**	0.12	-0.07
LL[e]	-0.09	0.46	**0.85**
LW[f]	**0.57**	-0.30	0.36
SPP[g]	**0.92**	-0.03	-0.11
Eigenvalue	2.73	1.35	0.98
Total variance (%)	38.94	19.24	13.99
Cummulative (%)	38.94	58.18	72.17

[a]Vine length (m) ; [b]Stem diameter (mm); [c]Number of leaves; [d]Leaf length (cm); [e]Leaf width (cm);[f]Number of branches on main stem; ; [g]Number of stem per plant.

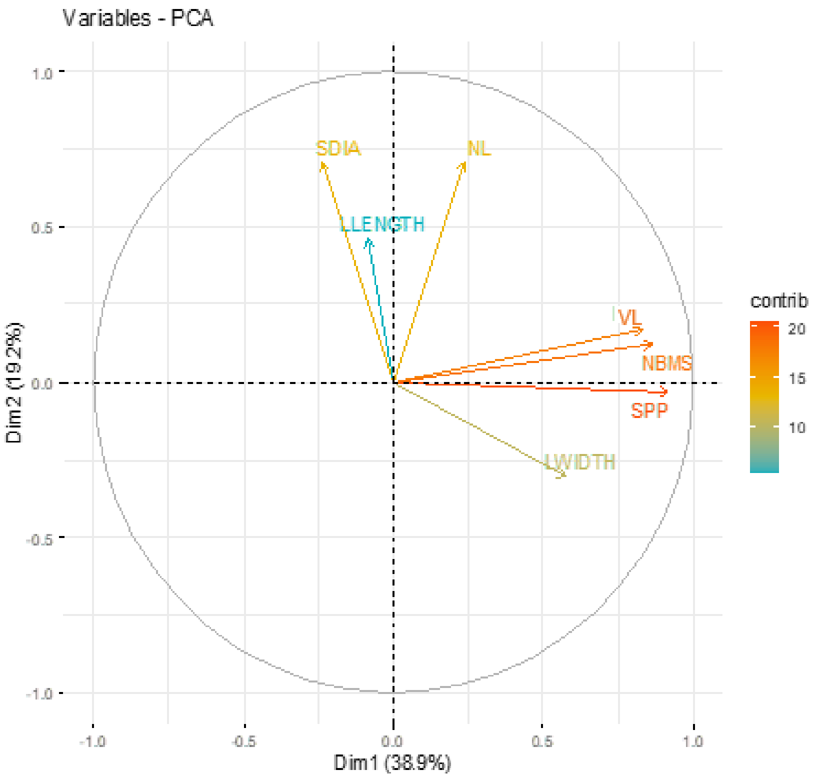

Figure 1: PCA plot showing the total contribution of variables accounting for variability in PC1 and PC2. VL:Vine length; NBMS: Number of branches on main stem of plants; SPP: Number

of stems per plant; Lwidth: Leaf width; LL: Leaf length; NL: number of leaves per plant; SDIA: Stem diameter.

Correlation analysis revealed positive relationship among the evaluated trait. In fact, the vine length displayed positive corelation with the stem per plant ad with the number of the branches per stem. Also, and through the correlation analysis positive colleration was reported between the leaf length, the number of branches per plant, the stem number per plant and the vine length while negative correlation was observed between the stem diameter and the number of stem per plant (Figure 2)

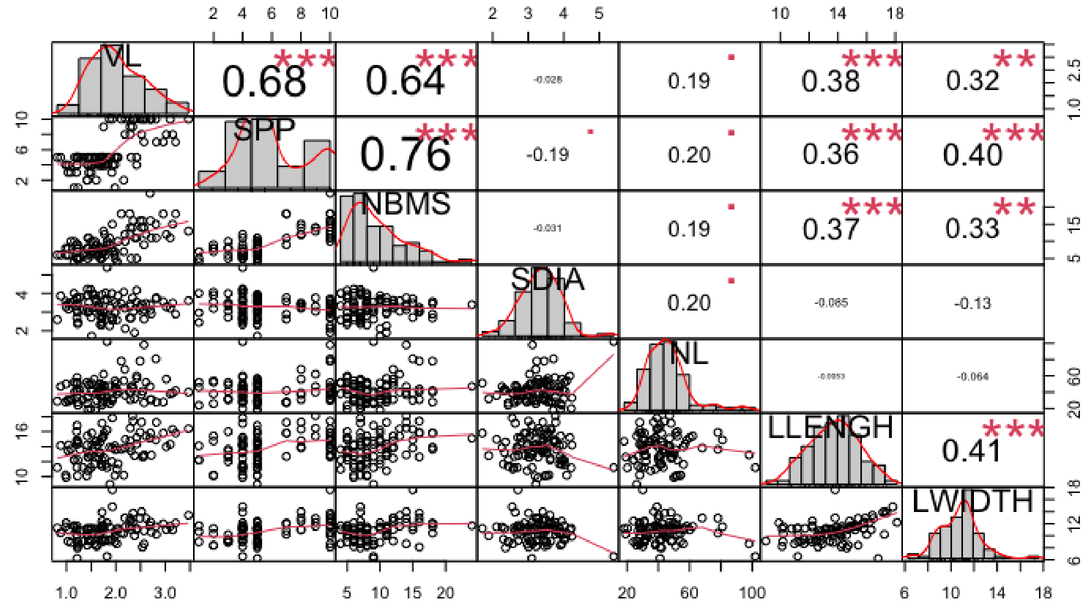

Figure 2: Phenotypic correlation among the evaluated quantitative traits

Using heatmap analysis, and based the agronomic traits, the 94 accessions were grouped into three cluster (Figure 3). Cluster 1 has the list accessions six in total and characterized by high biomass density (number of leaf) (Figure 3). The cluster 2 has 34 accessions and characterized by low leaf density combined with low vine length (Figure 3). Cluster 3 has the highest number of accessions (54 in total) and characterized by average leaf density (Figure 3)

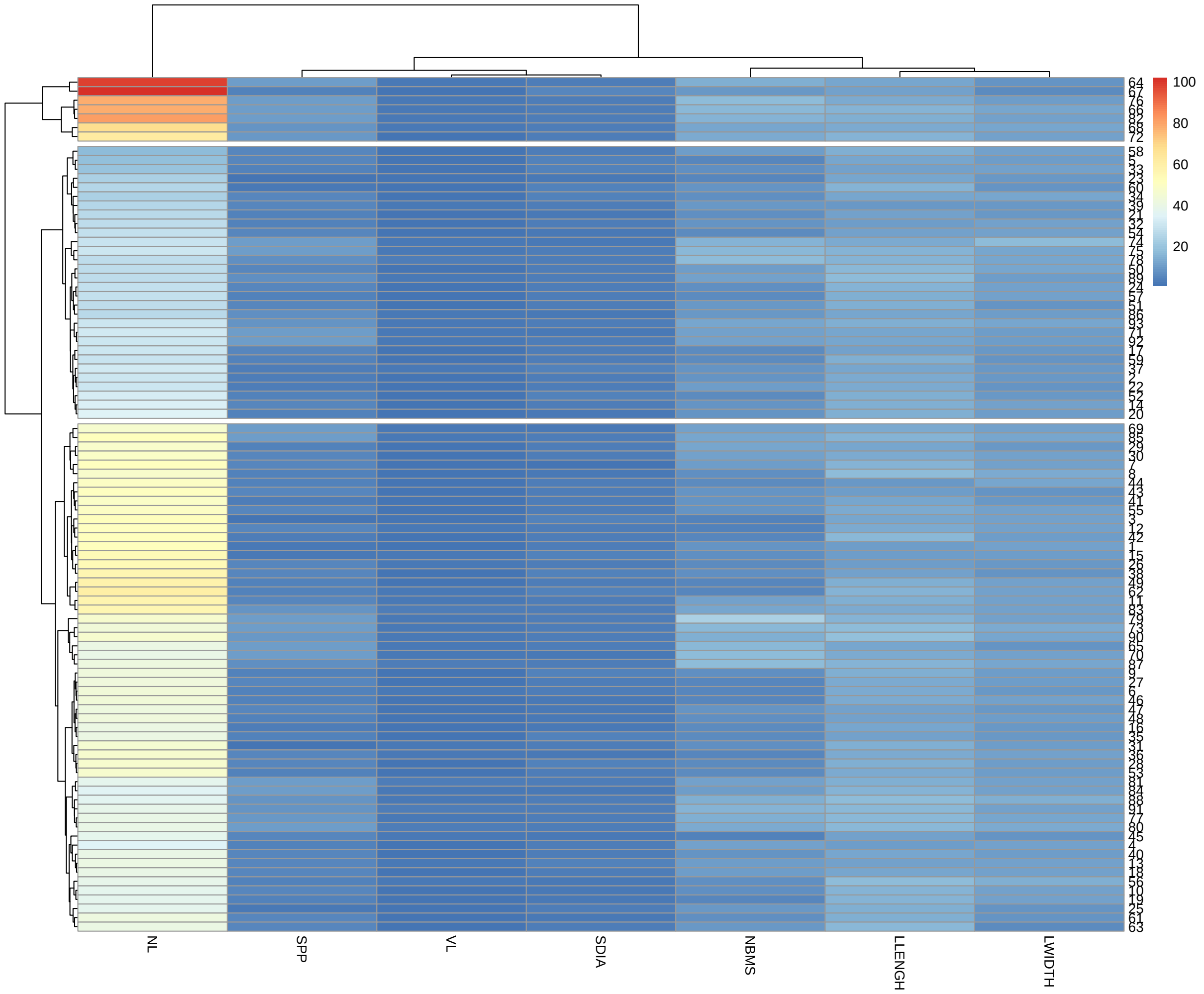

Figure 3: Heatmap classification based quantitative traits

Summary statictic based SNP markers

A total of 11,721 unfiltered SNP markers was initially generated, from which 10,087 was retained after the removal of low-quality SNP markers. The SNP markers were unequally distributed across the twenty (20) chromosomes of *D. bulbifera* (Table 5). The highest number of SNPs (516) was mapped on chromosome 10 and the lowest number of SNPs (489) was mapped on chromosome 15 (Table 5). Transition SNPs (62.39%, 6293 SNPs) were more frequent than transversions SNPs (37.61%, 3794 SNPs). The C/T transitions (31.14%) accounted for the highest frequency, while C/G transversions (6.77%) occurred at the lowest frequency (Figure 4). The average polymorphic information content (PIC) value across all the markers was 0.268, it ranged from 0.262 to 0.277 while the observed heterozygosity (Ho)

ranged from 0.252 to 0.278 with an average of 0.262 (Table 5, Figure 5). The expected heterozygosity (He) ranged between 0.320 and 0.342, and the mean genetic diversity was 0.329. Similarly, the minor allele frequency (MAF) ranged between 0.226 and 0.249 with an average of 0.235 (Figure 5).

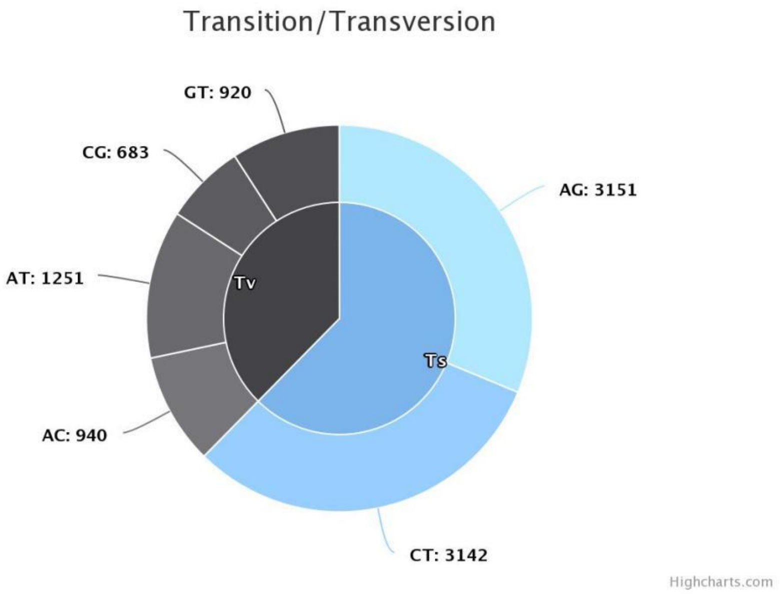

Figure 4: Transition and transversion based on bi-allelic SNP markers. Tv: Transversions; Ts: Transitions; A: Adenine; T: Thymine; G: Guanine; C: Cytosine. Chart developed using SNIPLAY software

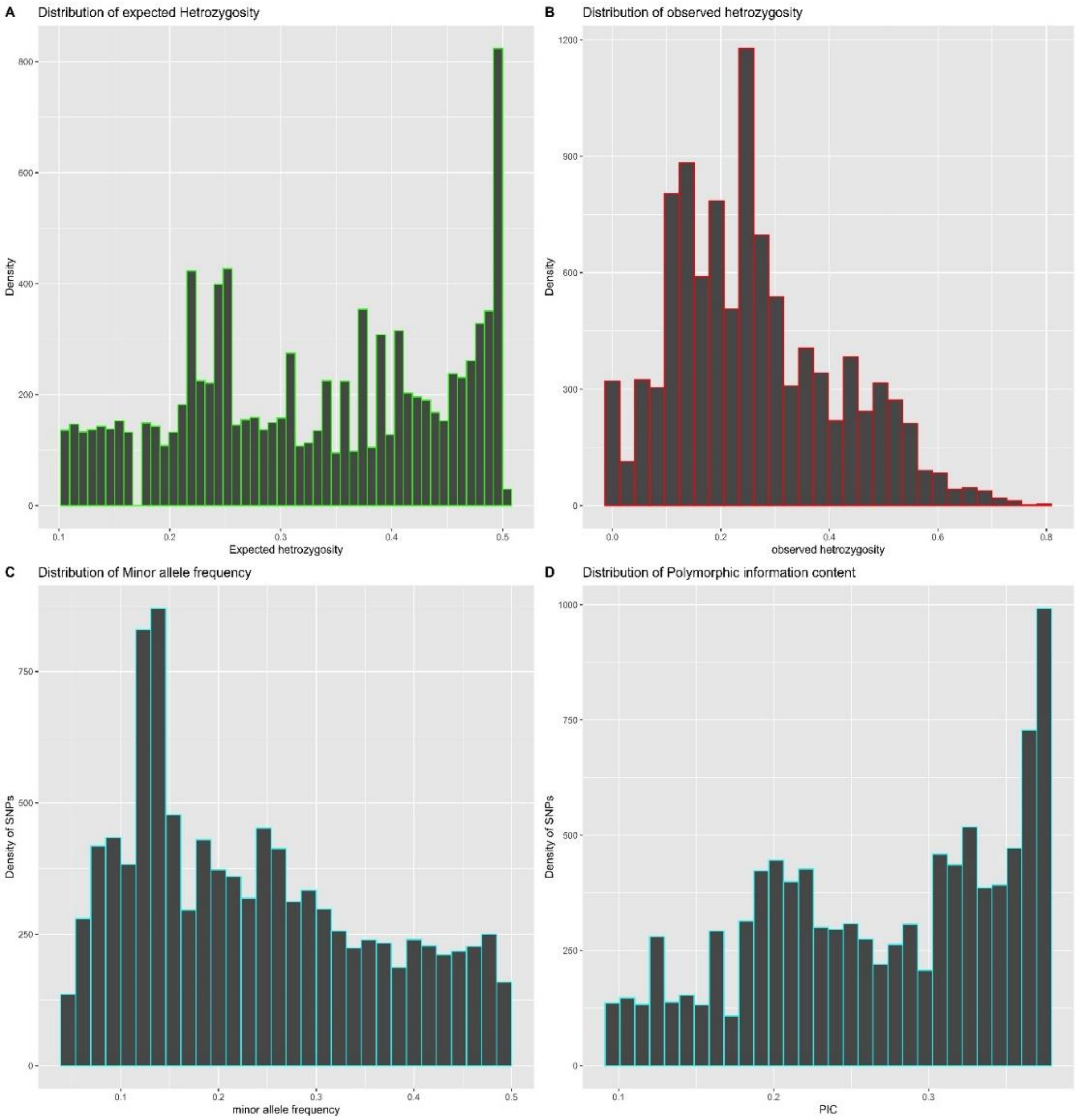

Figure 5: Histogram showing the distribution of SNPs markers associated with *D.bulbifera* genome. A) Distribution of expected heterozygosity in the genome B) Distribution of observed heterozygosity in the genome. C) Distribution of Minor allele frequency in the genome. D) Distribution of polymorphic information content.

Table 5: Summary statistics of SNP markers across 20 chromosomes of *D. bulbifera* accessions.

Chr	SNP no	Ho	He	PIC	MAF
1	511	0.268	0.342	0.277	0.249

2	509	0.262	0.330	0.269	0.235
3	490	0.257	0.326	0.266	0.232
4	509	0.264	0.325	0.265	0.233
5	511	0.257	0.323	0.264	0.227
6	507	0.268	0.328	0.267	0.233
7	507	0.271	0.337	0.273	0.243
8	506	0.278	0.330	0.268	0.237
9	507	0.256	0.320	0.262	0.226
10	516	0.257	0.322	0.263	0.229
11	514	0.271	0.328	0.267	0.233
12	494	0.262	0.325	0.265	0.231
13	492	0.256	0.330	0.269	0.233
14	513	0.256	0.329	0.268	0.235
15	489	0.263	0.340	0.276	0.242
16	505	0.267	0.332	0.270	0.237
17	510	0.257	0.334	0.271	0.239
18	510	0.255	0.324	0.264	0.228
19	498	0.252	0.326	0.266	0.232
20	509	0.267	0.333	0.270	0.242
Total/Average	10,087	0.262	0.329	0.268	0.235

SNP: Single nucleotides polymorphism; HO: Observed heterozygosity; He: Expected heterozygosity; PIC: Polymorphic information content; MAF: Minor allele frequency.

Population structure

Through the Bayesian Information Criterion (BIC) and complementary coordination analysis, three clusters was identified as the optimum number of the possible group number (Figure 6). The 94 accessions were classified into three (3) main groups (Figure 7) through phylogenic analysis. The first cluster (red), which were all made up of IITA genebank accessions. A larger number of accessions were in the second cluster (green) containing forty five (45) accessions

(47.87%) of which 40 were IITA genebank accessions and 5 were different accessions obtained from different communities within Anambra State of Nigeria. The third Cluster (blue) was made of twenty six (26) accessions (27.66%) which comprised mainly of the accessions obtained from other different south eastern states of Nigeria and one (1) IITA genebank accession (Figure 7). Through the admixture analysis, most of the accessions were properly assigned to a genetic group and only a few were considered as admixt (Figure 8). Out of the 94 accessions, only seven accessions (TDB 3045, TDB 3087, TDB 3049, TDB 3835, TDB 3119, TDB 3695) displayed ancestry probability < 50% and were considered as admixt. Simulations (logarithm probability relative to standard deviation, ΔK) estimated from the SNP markers showed a sharp peak at K=3 which explained the optimum number of sub-populations (ΔK=3). At ΔK = 3, cluster I, cluster II and cluster III consisted of 26 accessions (27.66%), 45 accessions (47.87%), and 23 accessions (24.47%) respectively.

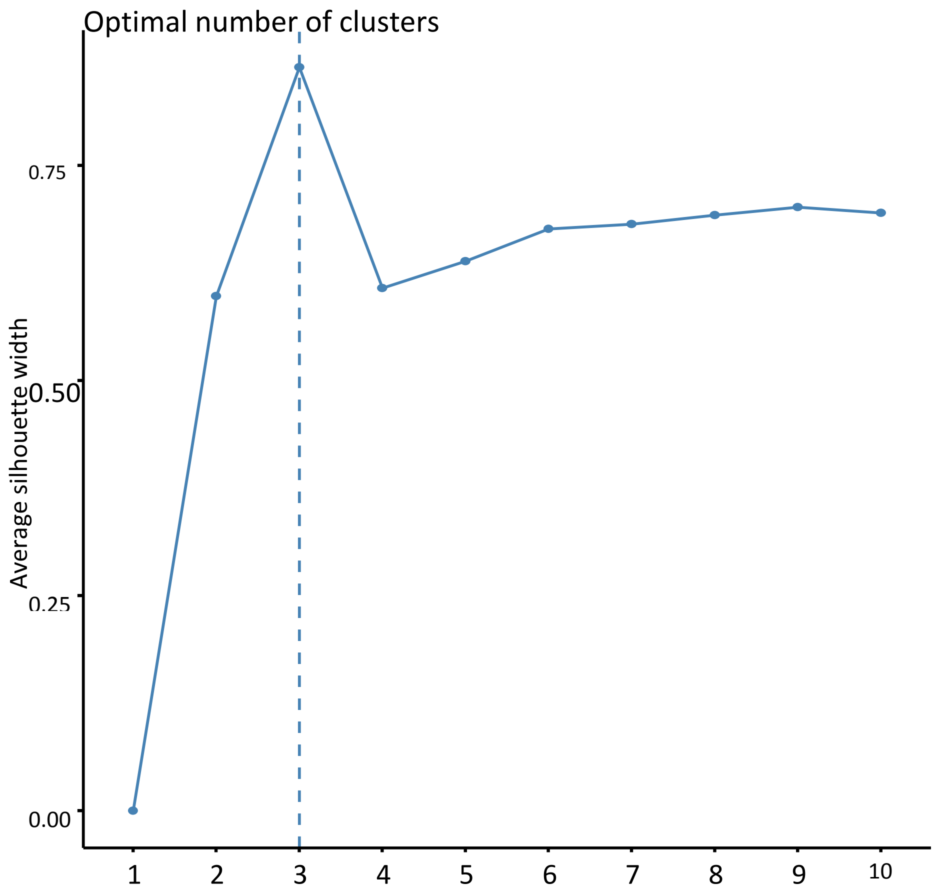

Figure 6: Graph representing the estimated membership fraction using LnP-(D) derived delta K with clusters number (K) ranged from 1-10 for K=3

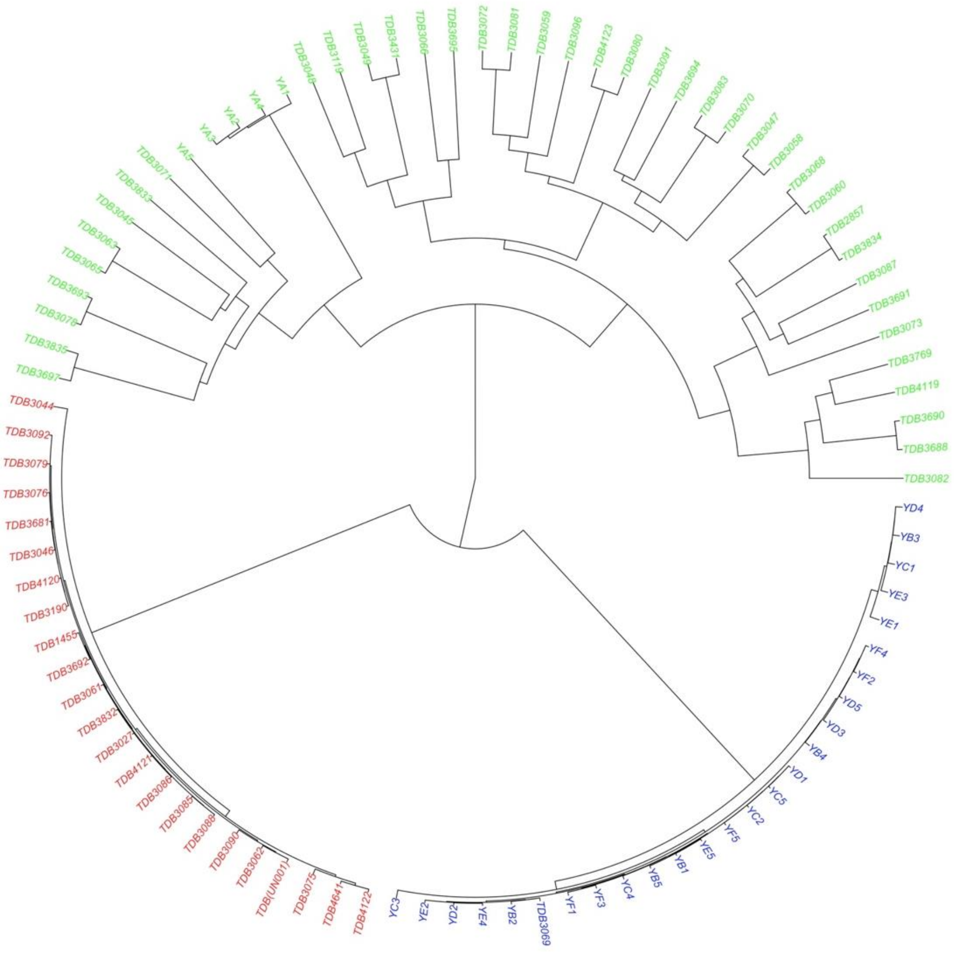

Figure 7: Hierarchical circular clustering dendrogram generated using the UPGMA method and Jaccard's dissimilarity matrix. Different colours indicate different groups identified: Cluster 1 (red), Cluster 2 (green), Cluster 3 (blue).

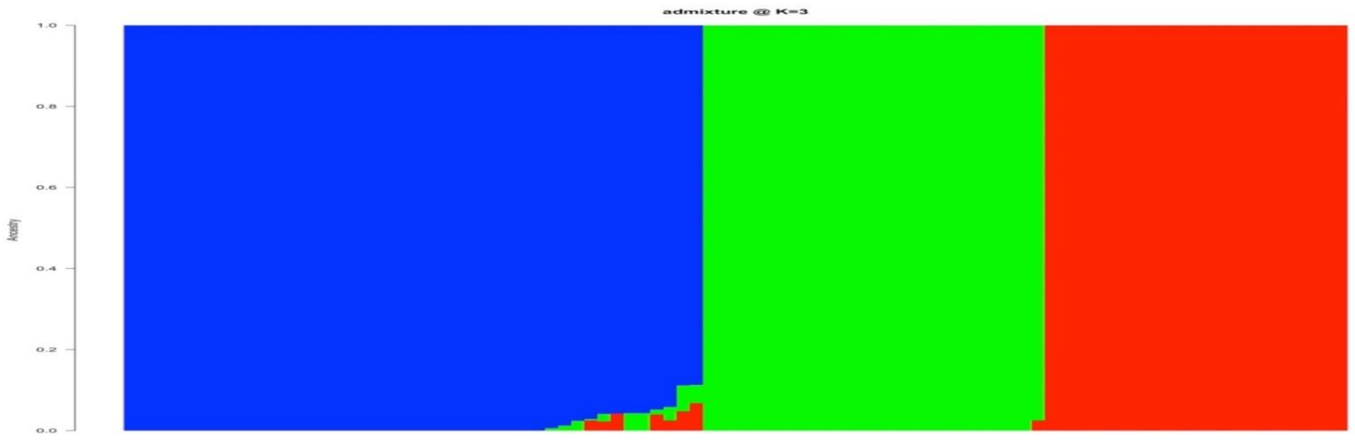

Figure 8: Grouping pattern in 94 accessions of *D. bulbifera* at K=3 based on the Bayesian clustering method. The colour displays each cluster: Blue (cluster 1), Green (cluster 2) and Red (cluster 3). Each vertical bar corresponds to an accession and the colour proportion in each bar represents the probability of each accession being affiliated to the different clusters

Discussion

In this study, the level and extent of genetic diversity among 94 accessions of *D.bulbifera* was dissected using both phenotypic and molecular markers. The results of the phenotypic quantitative traits used to assess variability in the yam genetic resources were successful in distinguishing the accessions based on their relationships or groupings. A better understanding of the existing aerial yam germplasm is one of the prerequisites for breeding new genotypes with novel or improved characteristics. Our study appeared very informative as high genetic variability was observed among the accessions studied. We deployed the use of standard agronomic and phenotypic evaluation developed by Asfaw [36] to the access variations and distinctions among *D. bulbifera* genotypes in IITA and NRCRI breeding programs in Nigeria. Obtained results indicate significant variation in genotypes evaluated thus providing the benchmark for optimising breeding protocols. Results indicated the presence of a high degree of agro-morphological polymorphisms among the accessions, pointing to possibilities of obtaining desirable trait combinations in specific accessions. Similar significant variations results have been reported by Osuagwu [48-50] in their works on the morphological diversity of *D. bulbifera* accessions. The principal component derived from studied quantitative traits (72.17%) coupled with large differences between minimum and maximum values of studied traits confers the strength of these traits in distinguishing studied cultivars. It equally suggests that the variability observed can be exploited for crop improvement. These variations is expected due to the agro ecological and country diversity of the initial collection sites of these materials. Observed variability could be as a result of sexual recombination [51], and natural mutation and long term selection occurring in the course of its ongoing domestication process in some agro-ecological zones [52]. Despite the potential of phenotypic traits in diversity studies, their expression may be partly subjected to environmental variation, thus, providing limited genetic information [18].

The DArTseq genotyping detected a total of 10,087 informative SNPs, which were unequally distributed among and within the 20 aerial yam chromosomes at various densities. The average PIC value of 0.27 obtained in the present study is higher than previous reports [53] but comparable to some other studies [54,55]. This shows the informativeness of the SNP markers used in this study. Other genetic diversity parameters such as minor allele frequency, observed

and expected heterozygosity levels displayed low variability from one region to another. This could be due to the presence of natural gene flow among the study zones and across countries.

Through structure and phylogenetic tree analyses, the results displayed a non-random distribution of alleles and accessions. The 94 aerial yam accessions used in this study were classified into three subgroups. For all the subgroups, both accessions obtained from IITA genebank and the eastern part of Nigeria were well distributed and showed high correspondence in clustering patterns between the different grouping methods. Similar results were observed in *Dioscorea alata* [35] and cowpea [56]. The three subgroups of aerial yam accessions was observed to exhibit a low level of admixture. These observed genetic divergence with a low admixture level is due to original differences in domestication and subsequent vegetative propagation by farmers. Yam clones in farmer's field are genetically homogenous with negligible recombination rates, as farmers select their planting material from tubers and not from botanical seed. However, the molecular evidence on ennobled cultivars showed that the tubers collected by farmers from wild environments are often a mixture of wild and interspecific hybrids [57,58,40]. This could partly explain the origin of the genetic admixture identified among the aerial yam accessions.. Harnessing the advantages of phenotypic and molecular markers improves the grouping of entries in a germplasm collection [59], which provides a piece of valuable base information for parental selection to realize and sustain genetic gain. Results of the diversity status of *Dioscorea bulbifera* yam genotypes derived from IITA genebank and NRCRI breeding programs using high-throughput genotyping approach (DArTseq) identified 10,087 SNP markers distributed across the 20 chromosomes as observed in Agre *et al*.,[34,32]. The SNP markers exhibited polymorphic values with an average of 0.27. This is similar to trends reported by previous studies on yam [31,32,53] in *D.alata*. To our knowledge, our study represents the first high throughput genotyping of *D. bulbifera* and thus highlights the valuable prospect of utilizing DArTseq-SNP markers for molecular genetic studies in yams. Our findings buttress the fact that there are in and across country related variations. The heterozygosity (He) values recorded in this study affirms the view of relatively high heterozygosity among genotypes when compared with similar studies [31,32,53]. The studied genotypes were collected from different countries and diverse locations in Nigeria. The selected SNP markers resulted to three cluster groups. Two independent cluster groups comprised mainly individuals from IITA genebank while a third cluster comprised mostly of cultivars obtained from South Eastern states of Nigeria. Using DAPC, the three

clusters exhibited genetic differentiation with significant variations. The trend in variability infers evolutionary and admixture relationship as reported by Adjei [31]. Cluster II had individuals from different countries suggesting some sort of clonal dispersion though trade and migration within the African sub region. This implies the existence of a robust genepool from which breeding programs can thrive to make selection and breeding advance. It is noteworthy to highlight that breeding programs on yam has prioritised *D. rotundata* and *D.alata* in the past. This conscious effort sets the very foundation for *D. bulbifera* crop improvement strategy.

Conclusion

In this study, we made used of agronomic traits and DArTseq markers to assess the genetic diversity. High variability was obtained among the evaluated accessions based on their agronomic performance. Population structure and hierarchical clustering analysis the accessions in three distinct genetic groups with high genetic variability among genetic group. Based on the result, the diversity could serve as valuable sources of novel genes for *D. bulbifera* improvement.

Author contributions

EE, EN, PO, PAA and JO conceptualized the study idea. EE, JO, PAA , assembled the genetic materials. EE, PAA, KE coordinated data generation. EE, IN, PAA analysed the data. EE, JO, PAA wrote the drafts. All authors reviewed the manuscript and approved the submitted version.

Ethics statement

The study was conducted in the view of profiling *D. bulbifera* for breeding purpose. All activities was conducted in respect of the study design. All authors reviewed the manuscript and approved the submitted version.

Funding

The funding support from core budget of Yam breeding program from the BMGF, National Root Crops Research Institute (NRCRI) Umudike, Nigeria is acknowledged.

Acknowledgments

We are grateful for the technical support the yam breeding team of NRCRI provided in field data collection. We acknowledge the yam team of the IITA Bioscience center for their laboratory support

Conflict of interest

The authors declare no conflict of interest.

REFERENCES

1. Ayensu, E. S. and Coursey, D. G. 1972. Guinea yams: The botany, ethnobotany, use and possible future of yams in West Africa. *Economic Botany* 26.4: 301−318.
2. Govaerts, R., Wilkin, P. and Saunders, R. M. K. 2007. *World checklist of Dioscoreales yams and their families*. Kew Publishing, Royal Botanic Gardens, Kew, United Kingdom. xviii + 65pp.
3. Asiedu, R. and Sartie, A. 2010. Crops that feed the World 1. Yams. Food Secur., 2, 305–315.
4. Nweke FI, Aidoo R, Okoye B, 2013. Yam consumption patterns in West Africa. Final Report for Bill and Melinda Gates Foundation (BMGF).
5. Lebot, V. (2009). Tropical root and tuber crops: cassava, sweet potato, yams and aroids. In: Pests and diseases. (Lebot V, eds.). CAB International, Wallingford, 253-264
6. Obidiegwu JE, Akpabio EM, 2017. The geography of yam cultivation in southern Nigeria: Exploring its social meanings and cultural functions. *J. Ethn. Foods,* 4: 28-35.
7. Obidiegwu JE, Lyons JB, Chilaka CA, 2020. The *Dioscorea* genus (yam)- An appraisal of nutritional and therapeutic potentials. *Foods,* 9:(1304) 1-45.
8. FAO, 2021. Food and Agriculture Organization of the United Nations, FAOSTAT statistical database. Rome.
9. Thottappilly, G., Mignouna, H. D., Onasanya, A., Abang, M., Oyelakin, O. and Singh, N. K. 1999. Identification and differentiation of isolates of *Colletotrichum gloeosporioides* from yam by random amplified polymorphic DNA markers. *African Crop Science Journal* 7 .2: 195−205.
   African Journal of Biotechnology 8 (12) 8(12), 2730-2739.
10. Winch, J. E., Newhook, F. J., Jackson, G. V. H. and Cole, J. S. 1984. Studies of *Colletotrichum gloeosporioides* disease on yam, *Dioscorea alata,* in the Solomon Islands. *Plant Pathology* 33: 467−477.
11. Hahn, S. K., Osiru, D. S. O., Akoroda, M. O. and Otoo, J. A. 1987. Yam production and its future prospects. *Outlook on Agriculture* 16.3: 105−110.

12. O'Hair, S. K. 1984. Farinaceous crops. *Handbook of Tropical Food Crops*. F. W. Martin (editor). CRC Press, Inc. Boca Raton, Florida. 109−138.
13. Eka, O. U. 1985. The chemical composition of yam tubers. *Advances in yam research. The biochemistry and technology of yam tubers*. G. O. Osuji (editor). Volume 1. The Biochemical Society of Nigeria in Collaboration with Anambra State University of Technology, Enugu, Nigeria. Pp. 51−75.
14. Coursey, D. G. 1976. The origins and domestication of yams in Africa. In: *Origins of African plant domestication*. J. R. Harlan, J. M. J. De Wet, and A. B. L. Stemler (editors). Mouton and Co. The Hague. Pp. 383−408.
15. Eka, O. U. 1998. Roots and tubers. *Nutritional quality of plant foods*. A. U. Osagie and O. U. Eka (editors). Post Harvest Research Unit, Department of Biochemistry, University of Benin, Benin City, Nigeria. Pp. 1−31.
16. Kundu B, B., Vanni K, Farheen A, Jha P, Pandey D.K, Kumar V. 2021, *Dioscorea bulbifera* L. (Dioscoreaceae): A review of its ethnobotany, pharmacology and conservation needs, South African Journal of Botany, 140, 365-374,https://doi.org/10.1016/j.sajb.2020.07.028.
17. Mulualem, T.; Mekbib, F.; Shimelis, H.; Gebre, E.; Amelework, B. 2018. Genetic diversity of yam (*Dioscorea* spp.) landrace collections from Ethiopia using simple sequence repeat markers. Aust. J. Crop Sci 12, 1223–1230.
18. Tamiru, M.; Heiko, C.; Becker, H.C.; Maass, B.L. 2011. Comparative analysis of morphological and farmers' cognitive diversity in yam landraces (*Dioscorea* spp.) from Southern Ethiopia. Trop. Agric. Dev. 55, 28–43.
19. Satya, P.; Karan, M.; Jana, S.; Mitra, S.; Sharma, A.; Karmakar, P.G.; Ray, D.P. 2015. Start codon targeted (SCoT) polymorphism reveals genetic diversity in wild and domesticated populations of ramie (Boehmeria nivea L. Gaudich.), a premium textile fiber producing species. Meta Gene. 3, 62–70.
20. Terauchi, R., Terachi, T. and Tsunewaki, K. 1991. Intraspecific variation of chloroplast DNA in *Dioscorea bulbifera* L. Theor. Appl. Genet. 81: 461-470.
21. Ramser, J., Weising, K., Kahl, G., López-Peralta, C. and Wetzel, R. 1996. Genomic variation and relationships in aerial yam (*Dioscorea bulbifera* L.) detected by random amplified polymorphic DNA. Genome 39: 17-25.
22. Dixit, S., Mandal, B. B., Ahuja, S. and Srivastava, P. S. 2003. Genetic stability assessment of plants regenerated from cryopreserved embryogenic tissues of *Dioscorea bulbifera* l. Using RAPD, biochemical and morphological analysis. Cryo Lett. 24: 77-84.
23. Narula, A., Kumar S., Bansal, K. C. and Srivastava, P. S. 2003. In vitro micropropagation, differentiation of aerial bulbils and tubers and diosgenin content in *Dioscorea bulbifera*. Planta Med. 69: 778-779.
24. Tamiru, M., Becker, H. C. and Maass, B. L. 2007. Genetic diversity in yam germplasm from Ethiopia and their relatedness to the main cultivated *Dioscorea* species assessed by AFLP markers. Crop Sci. 47: 1744-1753.
25. Obidiegwu JE , Asiedu R , Ene-Obong EE, Muoneke CO 2009a. Genetic characterization of some water yam (Dioscorea alata L.) accessions in West Africa with simple sequence repeats. ournal of Food, Agriculture & Environment 7 (3&4), 634-638

26. Obidiegwu JE, Kolesnikova-Allen M, Ene-Obong EE, Muoneke CO , Asiedu R. 2009b. SSR markers reveal diversity in Guinea yam (Dioscorea cayenensis/D. rotundata) core set...

27. Yan, Q. Q., Li, Y., Sun, X. Q., Guo, J. L., Hang, Y. Y. and Li, M. M. 2014. Isolation and characterization of polymorphic microsatellite loci from aerial yam (*Dioscorea bulbifera* L.). Genet. Mol. Res. 13: 1514-1517.

28. Croxton, M. D., Andreu, M. A., Williams, D. A., Overholt, W. A. and Smith, J. A. 2011. Geographic origins and genetic diversity of air-potato (*Dioscorea bulbifera*) in Florida. Invas. Plant Sci. Manage. 4: 22-30.

29. Girma, G., Hyma, K.E., Asiedu, R., Mitchell, S.E., Gedil, M., Spillane, C. 2014. Next-generation sequencing based genotyping, cytometry and phenotyping for understanding diversity and evolution of guinea yams. Theor. Appl. Genet., 127, 1783–1794.

30. Agre, P.; Norman, P.E.; Asiedu, R.; Asfaw, A. 2021. Identification of Quantitative Trait Nucleotides and Candidate Genes for Tuber Yield and Mosaic Virus Tolerance in an Elite Population of White Guinea Yam (*Dioscorea rotundata*) Using Genome-Wide Association Scan. BMC Plant Biol. 21, 552.

31. Adjei E A, Esuma W, Alicai T, Bhattacharjee R, Dramadri IO, Edema R, Chamba E B, Odong TL.v2023. Genetic diversity and population structure of Uganda's yam (Dioscorea spp.) genetic resource based on DArTseq. PLoS ONE 18(2) doi.org/10.1371/journal.pone.0277537

32. Agre, P. A., Edemodu, A., Obidiegwu, J. E., Adebola, P., Asiedu, R. and Asfaw, A. 2023. Variability and genetic merits of white guinea yam landraces in Nigeria. Front Plant Sci. 14:1051840

33. Cormier, F., Mournet, P., Causse, S., Arnau, G., Maledon, E., Gomez, R. M., Pavis, C. and Chair, H. 2019. Development of a cost effective single nucleotide polymorphism genotyping array for management of greater yam germplasm collections. Ecological Evolution. 9, 5617–5636

34. Agre, P., Asibe, F., Darkwa, K., Edemodu, A., Bauchet, G., Asiedu, R., Adebola, P. and Asfaw, A. 2019. Phenotypic and molecular assessment of genetic structure and diversity in a panel of winged yam (*Dioscorea alata*) clones and cultivars. Sci. Rep. 9, 1-11.

35. Siadjeu, C., Mayland-Quellhorst, E. and Albach, D.C. (2018). Genetic diversity and population structure of trifoliate yam (*Dioscorea dumetorum* Kunth) in Cameroon revealed by genotyping-by-sequencing (GBS). BMC Plant Biol., 18, 359

36. Asfaw, A. 2016. Standard operating protocol for yam variety performance evaluation trial IITA Ibadan Nigeria 27, 1–33.

37. DOYLE, J.J.; DOYLE J.L 1990. Isolation of plant DNA from fresh tissue. Focus.12,.13-15

38. Aljanabi SM, Martinez I. 1997. Universal and rapid salt-extraction of high quality genomic DNA for PCR-based techniques. Nucleic Acids Research . 25(22):4692-3. doi: 10.1093/nar/25.22.4692.

39. Kilian A, Sanewski G, Ko L. 2016. The application of DArTseq technology to pineapple. Acta Horticulture DOI:10.17660/.1111.27

40. Sugihara Y, Darkwa K, Yaegashi H, Natsume S, Shimizu M, Abe A, Hirabuchi A, Ito K, Oikawa K, Muluneh T, Ohta A, Matsumoto R, Agre P, De Koeyer D, Pachakkil B, Yamanaka S, Muranaka S, Takagi T, White B, Asiedu R, Innan H, Asfaw A, Adebola P and Terauchi R. 2020. Genome analyses reveal the hybrid origin of the staple crop white Guinea yam (*Dioscorea rotundata*). Proceedings of the National Academy of Sciences 50, 31987–31992.

41. Purcell, S., Neale, B., Todd-Brown, K., Thomas, L., Ferreira, M.A.R., Bender, D., Maller, J., Sklar, P., de Bakker, P.I.W., Daly, M.J., and Sham, P. C. 2007. PLINK: A tool set for whole-genome association and population-based linkage analyses. Am. J. Hum. Genet, 81, 559–575.

42. Danecek, P., Auton, A., Abecasis, G., Albers, C.A., Banks, E., DePristo, M.A., Handsaker, R.E., Lunter, G., Marth, G.T., Sherry, S.T., McVean, G. and Durbin, R. 2011. The variant call format and VCFtools. Bioinformatics, 27, 2156–2158.

43. Lê, S., Josse, J. and Husson, F. 2008. FactoMineR: an R package for multivariate analysis. J. Stat. Soft. 25: 1- 18.

44. Jeffers J. N. 1967. Two case studies in the application of principal component analysis. J. Roy. Stat. Soc: Ser. C (Appl. Stat.) 16: 225-236.

45. Parida, S.K., Mukerji, M., Singh, A.K., Singh, N.K. and Mohapatra, T. 2012. SNPs in stress-responsive rice genes: Validation, genotyping, functional relevance and population structure. BMC Genom. 13, 426.

46. Nimmakayala, P., Levi, A., Abburi, L., Abburi, V.L., Tomason, Y.R., Saminathan, T., Vajja, V.G., Ma, S.A., Reddy, R., Wehner, T.C., Mitchell S. E. and Reddy, U. K. 2014. Diversity, linkage disequilibrium, and selective sweeps in cultivated watermelon. BMC Genom., 15, 767.

47. Uribe-Salazar, J. M., Palmer, J. R., Haddad, S. A., Rosenberg, L., & Ruiz-Narváez, E. A. 2018.. Admixture mapping and fine-mapping of type 2 diabetes susceptibility loci in African American women. *Journal of human genetics*, *63*(11), 1109-1117.

48. Osuagwu, A. N., Edem , U. L. and Kalu , S. E. 2019. Evaluation of genetic diversity in aerial yam (*Dioscorea bulbifera* L) using qualitative and quantitative morphological traits. *Global Scientific Journal*. Vol 7, 11: 2320-9186.

49. Jayeola, AA. , Oyebola, T.O. (2013). Morpho-molecular studies in the natural populations of Dioscorea bulbifera Linn in Nigeria. Journal of Genetics and Molecular Biology, 14: 1061- 112

50. Beyene, T. M. 2013. Genetic diversity of aerial yam (Dioscorea bulbifera. L) Accessions in Ethiopia based on agronomic traits. Agriculture, Forestry And Fishery, 2(2): 67-71

51. Tostain S., Agbangla C., Scarcelli N., Mariac C., Dainou O., Berthaud J., Pham J.L. 2007. Genetic diversity analysis of yam cultivars (*Dioscorea rotundata* Poir.) in Benin using simple sequence repeat (SSR) markers. Plant Genetic Resources. 5, 71–81

52. Kouam, E B, Avana-Tientcheu M L., Lekeumo, V D., Akitio H M., Khasa D P, Pasquet R S. 2018. Agro-ecological distribution of the phenotypic diversity of aerial yam (*Dioscorea bulbifera* L.) in Cameroon using multivariate analysis: prospect for germplasm conservation and improvement. *Open Agriculture,* 3 (1), 190-206. https://doi.org/10.1515/opag-2018-0020

53. Bhattacharjee R, Agre P, Bauchet G, De Koeyer D, Lopez-Montes A, Kumar P L, Abberton M, Adebola P, Asfaw A, Asiedu R. 2020. Genotyping-by-Sequencing to Unlock Genetic Diversity and Population Structure in White Yam (Dioscorea rotundata Poir.) A*gronomy*, *10*(9), 1437; https://doi.org/10.3390/agronomy10091437

54. Simko I, Eujayl I, van Hintum TJ. 2012. Empirical evaluation of DArT, SNP, and SSR marker-systems for genotyping, clustering, and assigning sugar beet hybrid varieties into populations. Plant Science . 2012;184:54–62.

55. Zhang, W. J. , Chen, J. Y. , Liu, B. C. , Zhao, Y. Q. , Huang, Y. Z. , & Chen, Y. 2019. Genetic variations on major traits of 37 Chinese Yam germplasms. Fujian Journal of Agricultural Sciences, **34**(11), 1246–1254.

56. Sodedji FAK, Agbahoungba S, Agoyi EE, Kafoutchoni MK, Choi J, Nguetta SA, et al. 2021. Diversity, population structure, and linkage disequilibrium among cowpea accessions. Plant Genome.

57. Scarcelli N, Tostain S, Vigouroux Y, Agbangla C, Daïnou O and Pham JL. 2006. Farmers' use of wild relative and sexual reproduction in a vegetatively propagated crop. The case of yam in Benin. Molecular Ecology 9, 2421–2431.

58. Scarcelli N, Chaïr H, Causse S, Vesta R, Couvreur TL and Vigouroux Y. 2017. Crop wild relative conservation: wild yams are not that wild.Biological Conservation 210, 325–333.

59. Da Silva, J.A. 2017. The Importance of the wild cane *Saccharum spontaneum* for bioenergy genetic breeding. Sugar Tech 19: 229-240. DOI: 10.1007/s12355-017-0510-1
