# Supplementary material for: Exploring the genetic diversity and population structure of aerial yams (Dioscorea bulbifera L.) DArT-seq and agronomic traits
Source: PLoS One. 2024 Aug 23;19(8):e0306631. doi: 10.1371/journal.pone.0306631 (PMC11343425; doi:10.1371/journal.pone.0306631)
Supplement: Attachment — Submitted filename: Comments.docx [file pone.0306631.s002.docx]

Comment

The study on Exploring the Genetic Diversity and Population Structure of Aerial yams (Dioscorea bulbifera L.) DArT-seq and agronomic traits is a very extensive study which is accepted in the yam community as required to explore more genotypes for improvement study. However authors need to address the following to make the study scientifically sound for publishing.

**Generally,**

- All abbreviation should be checked and should be consistent throughout.

**Abstract:**

- This session is sound but will need some improvement pointing out some key results that are of significant importance to readers. Generalizing the results is not appropriate. Indicate some values and should be reelevate for the study.

**Introduction:**

- This session was simple and well written.

**Materials and methods:**

- **Phenotyping:** The use of just 7 variables seems not too extensive enough for the population in this study. Is there a reason for that? I suggest authors increase the number of variables by including flowering and some tuber characteristics. Remember that yam ontology has enough variable that can bring out the variation between these genotypes in the study.
- The variables codes were not consistent in the entire study**.** Authors should ensure that that variable codes are consistent.
- This will call for some figures and tables variable labels to be adjusted.
- Authors should add phenotypic clusters to the analysis of the phenotypic data.
- **Genotyping analysis:** Authors indicated AMOVA in the results, but the tools used for the AMOVA analysis were not indicated in the analysis section. Authors are expected to indicate that.

**Results:**

- This session needs more improvement.
- **Figures:** figures were not well cited in text. Some were not talked about completely and others were misplaced. I suggest authors should make it a point to check the figure very well.
- Clustering for phenotypic traits should be included in the results.
- The PCA Biplot can be improved by showing the variation of the genotypes based on source or origin or agroecological zone. This will make the plot very informative and appreciable.
- Summary genotypic statistics can be improved by indicating the Gene diversity and Call rate and other statistics to make table 5 very informative.
- Results on AMOVA were not presented in the results. A separate paragraph should be developed for that.
- The clustering was not explained well. I suggest a separate paragraph to be allocated for that.
- Note that the method of population structure using STRUCTURE, Clustering and AMOVA are different, and each should complement each other. Having them together does not make your store interesting for the study. I suggest authors provide separate paragraphs for STRUCTURE, AMOVA, CLUSTERING and if possible, include PCoA or PCA and indicate how these methods manage the population structure.
- More importantly, I suggest authors combine the results from phenotypic and genotypic to construct a cluster analysis to show the relation among them. That will be very good for this study.

**Discussion:**

- The discussion should be improved with any further analysis that is performed in the results section.
